# Application of machine learning for identification of heterotic groups in sunflower through combined approach of phenotyping, genotyping and protein profiling

**DOI:** 10.1038/s41598-024-58049-z

**Published:** 2024-03-27

**Authors:** Danish Ibrar, Shahbaz Khan, Mudassar Raza, Muhammad Nawaz, Zuhair Hasnain, Muhammad Kashif, Afroz Rais, Safia Gul, Rafiq Ahmad, Abdel-Rhman Z. Gaafar

**Affiliations:** 1 Crop Science Institute, National Agricultural Research Centre, Islamabad, Pakistan; 2 Colorado Water Centre, Colorado State University, Fort Collins, CO 80523 USA; 3 In-Service Agricultural Training Institute, Sargodha, Pakistan; 4 Department of Agricultural Engineering, Khwaja Fareed University of Engineering and Information Technology, Rahim Yar Khan, Pakistan; 5 Department of Agronomy, Arid Agriculture University, Rawalpindi, Pakistan; 6 Department of Botany, Sardar Bahadur Khan Women’s University, Quetta, Pakistan; 7 Barani Agricultural Research Institute, Chakwal, Pakistan; 8 Department of Botany and Microbiology, College of Science, King Saud University, 11451 Riyadh, Saudi Arabia

**Keywords:** Combining ability, Heterosis, Hierarchical clustering, K-means, Unsupervised clustering, Bioinformatics, Biological models, Computational models, Genome informatics

## Abstract

Application of machine learning in plant breeding is a recent concept, that has to be optimized for precise utilization in the breeding program of high yielding crop plants. Identification and efficient utilization of heterotic grouping pattern aided with machine learning approaches is of utmost importance in hybrid cultivar breeding as it can save time and resources required to breed a new plant hybrid/variety. In the present study, 109 genotypes of sunflower were investigated at morphological, biochemical (SDS-PAGE) and molecular levels (through micro-satellites (SSR) markers) for heterotic grouping. All the three datasets were combined, scaled, and subjected to unsupervised machine learning algorithms, i.e., Hierarchical clustering, K-means clustering and hybrid clustering algorithm (hierarchical + K-means) for assessment of efficiency and resolution power of these algorithms in practical plant breeding for heterotic grouping identification. Following the application of machine learning unsupervised clustering approach, two major groups were identified in the studied sunflower germplasm, and further classification revealed six smaller classes in each major group through hierarchical and hybrid clustering approach. Due to high resolution, obtained in hierarchical clustering, classification achieved through this algorithm was further used for selection of potential parents. One genotype from each smaller group was selected based on the maximum seed yield potential and hybridized in a line  ×  tester mating design producing 36 F_1_ cross combinations. These F_1_s along with their parents were studied in open field conditions for validating the efficacy of identified heterotic groups in sunflowers genetic material under study. Data for 11 agronomic and qualitative traits were recorded. These 36 F_1_ combinations were tested for their combining ability (General/Specific), heterosis, genotypic and phenotypic correlation and path analysis. Results suggested that F_1_ hybrids performed better for all the traits under investigation than their respective parents. Findings of the study validated the use of machine learning approaches in practical plant breeding; however, more accurate and robust clustering algorithms need to be developed to handle the data noisiness of open field experiments.

## Introduction

World population is expected to reach 9 billion by 2050, and provision of food, feed, and fiber to such a huge mass is the ultimate challenge for plant breeders to develop high yielding crop varieties^1^. Modelling of yield-growth trends has showed that increase in the yield of agricultural crops cannot cope with the increasing population, and the situation is getting more worst because of climate change particularly rising temperatures and extreme weather patterns^2^. During recent times, availability of different data sets, ranging from high throughput phenotyping to genotyping by SNPs (single nucleotide polymorphism) and resulting biochemical “omics” data sets are on rising side. However, the major challenge for “big data” of modern plant science and technology, is to efficiently and precisely predict phenotypes from underlying genotypes in changing climatic conditions. Variability in genetic/DNA sequences translated into bio-chemical outlook of the cells and tissues, and the bio-chemical makeup in collaboration with environmental cues transcribed into organ formation, plant growth and crop yield, and resistant to abiotic and biotic conditions. Under modern molecular plant breeding, exploring the impacts of environmental and genotypic variation opens new horizon regarding the regulation of essential process occurring in the life cycle of plant crops. They are also responsible for understating the quality traits and to predict the crop yield under particularly environmental situations^3^.

Analyzing phenotypes at the different levels of integrity and formation, devising links between phenotypes and genotypes require integration and processing of big, noisy, and heterogenous datasets. Machine learning (ML), a set of different statistical computation methods and approaches, can be used to discover various predictive patterns present in the dataset^4–6^. Machine learning-based product classification applications are powerful tools for developing accurate and robust classifiers. These applications include various algorithms such as decision trees, artificial neural networks, genetic algorithms, regression, and fuzzy logic. In addition, powerful algorithms are fruitful for developing diversified machine learning models, adjusting complicated input–output mapping approaches, choosing, and deleting the appropriate features. These models are frequently being applied to select the suitable and descriptive traits when assessing the quality of agricultural commodities^7^.

North America is considered the native region for sunflower and has captured the focus of agricultural scientists and farmers’ communities as one of the most essential industrial crops^8^. Regarding adaptive introgression and evolutionary biology, the *Helianthus* genus is enduring hybrid traits^9,10^. Sunflower has its own importance as it is considered in a model to track the sun’s direction. It is also helpful to understand the flower development processes in the plant science division^11^. Furthermore, sunflower crop is being cultivated throughout the world for its premium quality edible oil. At present, it is the fourth most important oilseed crop in the world. Reason for the widespread cultivation of sunflower is its ability to grow in wider range of environments, high seed yielding capacity, potential of having two crops in a calendar year^12,13^. To have cultivars with greater yield potential, it is established fact the F_1_s produced from distantly related high yield inbred lines are more transgressively superior to their parents or crosses attempted in closely related genotypes^14^.

ML applications have been playing a significant role in different engineering and medical related domains, but their true potential and usage in applied plant search is yet to be fully explored. In this present study, novel approach of utilizing three different machine learning approaches i.e., hierarchical clustering, k-means clustering, and a hybrid clustering (hierarchical + k-means) is utilized to identify heterotic patterns and then studied the practical application and efficacy of these machine learning based identified heterotic groups by developing F_1_ sunflower hybrids among heterotic groups.

## Materials and methods

### Experiment 1

#### Plant material

Experiment 1 was conducted in the National Agricultural Research Center (NARC), Islamabad which is situated on latitude 33.6641° N, and longitude 73.1276° E. Plant material comprised of 109 genetically diverse sunflower lines were used to mine grouping pattern and then efficacy of the identified grouping pattern in hybrid seed development in sunflower. Plant material (sunflower genotypes) was obtained from National Agricultural Research Centre, Islamabad, Pakistan (Table S1). Collection of plant materials complied with the institutional, national, and international guidelines and legislation. All experiment and analysis methods were performed following the relevant regulations and guidelines.

#### Phenotyping

To characterize the sunflower genotypes phenotypically, all the 109 sunflower lines were planted in open field conditions for two consecutive years in spring season following a randomized augmented block design. Data for nine plant morphometric characteristics i.e., plant height, stem curvatures, days to flower initiation, days to flower completion, number of leaves per plant, head diameter, leaf area, 100 seed weight and seed yield per plant were recorded from 10 randomly selected plants from each genotype, and their average was computed (Supplementary Table 1).

#### Genotyping

Molecular based genotyping of 109 sunflower lines was conducted by employing 40 SSR markers (Supplementary Table 2). Genomic DNA extracted from 1.5 to 2 weeks old seedlings of sunflower following cetyl trimethyl ammonium bromide (CTAB) method described by Saghai-Maroof et al.^15^. DNA was then diluted with 50 µl TE buffer and run on 1% agarose gel to determine its quality and concentration, before PCR analysis. DNA fragments amplified by the respective microsatellite marker were designated as a unit trait with 1 for the presence and 0 for the absence of a DNA band, thus generating a binary matrix data set.

#### Protein characterization (proteomic analysis)

Protein characterization was performed through SDS-PAGE of total seed proteins in vertical slabs as described by Jan et al.^16^. Separating and stacking gels were prepared with different concentrations. The gels were run on 100 V till the BPB marker reached the bottom of gels. A pre-standard protein ladder with a range of 10–180 kDa (Lot: 00345 035) was used to determine the molecular weights of sunflower seed proteins. Staining of gels was performed using 0.25% (w/v) Commasie brilliant blue (CBB) solution homogenized in 10% (v/v) acetic acid and 40% (v/v) methanol diluted in water, to visualize the proteins on gels. After staining gels were de-stained to wash away extra CBB dye. The de-staining solution contained 5% acetic acid (v/v), 20% methanol (v/v) and distilled water in 5:20:75 ratios.

Each polypeptide band in a gel corresponds to a unit character and designated as 1 for the presence of a band in a particular sunflower genotype under observation and 0 for the absence of protein band. Therefore, generated a binary matrix of proteins bands observed in 109 sunflower genotypes under study.

#### Machine learning baseline

**Figure 1:**
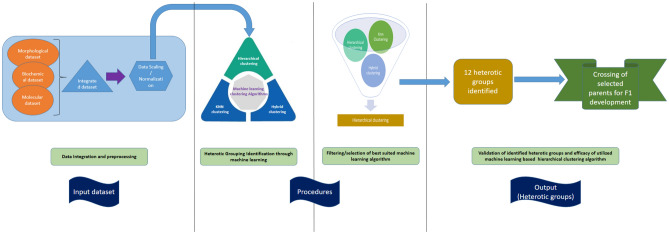
A schematic workflow of the procedures adopted to identify the underlaying heterotic grouping pattern in the studied germplasm pool of sunflower. The input dataset obtained was from the combination of phenotypic (9 morphological traits); genotypic (binary data obtained from 40 SSR markers scoring); proteomic (binary data from SDS-PAGE based characterization). The dataset was then scaled to the values ranging between 0 and 1 to remove data biasedness. Three machine learning based classifiers were then tested to produce the heterotic grouping in the studied sunflower materials. Out of three, Hierarchical clustering showed the maximum resolution power in terms of correctly classifying the A, B, R and SFP lines. Hierarchical clustering classified the 109 sunflower genotypes in 12 heterotic groups, and this clustering pattern was then used to develop F1 hybrids, while selecting 1 sunflower line from each heterotic group. Both hierarchical and Knn based machine learning clustering have been widely used in plants dataset (including phenotypic and genotypic data) for classification of plant genotypes.

The application of artificial intelligence in the pacing of the breeding of new cultivars is a burgeoning area of development considering the major impact it could create in the plant breeding field. The machine learning baseline^17^ is a generic, modular, and reusable workflow that combines agronomic principles of crop modeling with machine learning. The input data consists of a diverse aggregated dataset collected at three different levels of plant’s organizational structure i.e., morphological, molecular, and biochemical. The corresponding outputs is a set of clustering pattern of 109 sunflower genotypes obtained by the application of machine learning classifiers i.e., hierarchical clustering, K-means clustering and hybrid (hierarchical + K-means) clustering algorithms. A schematic workflow describing the interrelationship between input variables (integrated dataset) and the corresponding output (heterotic groups) using 3 machine learning qualifiers is presented in Fig. 1. Strategy followed for three different datasets collection, processing and integrations was as follows:

- Data from nine morphological parameters were collected from 109 sunflower genotypes.
- Genotyping data scores (binary format) was collected from amplification data of 40 SSR primers.
- Protein characterization data (binary format) was extracted from the amplification data of 14 different protein bands obtained after SDS-PAGE analysis of respective 109 sunflower genotypes.
- Collected data from all there sources (morphological, molecular, and biochemical) were scaled in a range of 0–1.
- Three different machine learning classifiers (hierarchical, K-means and hybrid (hierarchical + K-means) was employed for selection of suitable genotypes for F_1_ hybrid development and evaluation of identified heterotic groups in the studied genotypic pool of sunflower.
- The resultant classification pattern obtained from each machine learning classifier algorithm was evaluated for selection of suitable genotypes.
- Finally, performance of F1 hybrids was then evaluated to characterize the efficiency of machine learning algorithm in identification of suitable parental genotypes in hybrid breeding programs.

#### Data preparation and preprocessing

Data normalization and scaling is an important preprocessing procedure to remove data nuisance and improve the efficiency of learning from data^18^. Moreover, when different variables recorded have varied nature of recording i.e., continuous and binary, more care needs to be adopted to remove over/less fitting of the machine learning algorithms^19^. In this experiment, morphological data variables were recorded according to different scales, and the genotyping and protein profiling data were recorded in a binary scale. To address the overfitting/inaccuracy of machine learning algorithms, data scaling was performed according to Yeo and Johnson normalization method^20^. During data scaling, all traits were scaled to a [0,1] range using the following equation:

$${X}_{scaled}=\left[\frac{\left(X-{X}_{min}\right)}{\left({X}_{max}-{X}_{min}\right)} \times \left({X}_{max}-{X}_{min}\right)\right]+{X}_{min}$$

where ${X}_{scaled}$ is the scaled value of input variable, *X*_*min*_ and *X*_*max*_ are the minimum and maximum values of X variable, respectively. This scaling of data has also ensured that there are no outliers in the final dataset.

#### Data integration and heterotic grouping identification

For careful and accurate identification of heterotic grouping patterns present in the sunflower genotypes’ pool, all the three datasets, i.e., morphological, genotypic and proteins, were aggregated and evaluated collectively after data scaling. Three classification algorithms i.e., (a) hierarchical clustering, (b) K-means clustering and (c) hybrid clustering (hierarchical + K-means) was applied over the aggregated dataset. Data preprocessing, and application of machine learning algorithms were performed using R-Studio version 1.2.1335. Packages used for application of the above mentioned three machine learning algorithms were factoextra, FactomineR, dendextend, cluster and tidyverse.

Algorithms were compared and the one with highest resolution was selected for further selection of genotypes from the genotypic’ pool. The algorithm that classifies the sunflower genetic pool under study (A-lines, B-lines, R-lines) and self-pollinated (SFP) with more accuracy was regarded as the one with highest resolution power. Clustering pattern obtained from the best explaining algorithm by using aggregated dataset was then carefully evaluated to select the highly diverse and high yielding sunflower genotypes, i.e., one genotypes from each heterotic group (with the assumption that the selected genotype has the same breeding potential as the rest of genotypes in the same heterotic group). The selected genotypes were then crossed with each other to obtain F_1_ sunflower hybrids.

### Experiment 2

#### Evaluation of identified heterotic groups

##### F_1_ crosses development and evaluation

Based on the results of experiments 1, one genotype from each identified heterotic group was selected as a representative of whole group and utilized in hybridization scheme following a Line × Tester mating design. Each male line was crossed with each female line. 6 CMS lines identified were crossed with 6 Restorer lines to generate 36 sunflower F_1_ hybrids.

#### F_1_ phenotyping

Sunflower F_1_ hybrid obtained were tested in open field conditions at National Agricultural Research Center, and data regarding growth and yield attributes (days to flower initiation, days to flower completion, plant height, stem curvature, head diameter, number of leaves per plant, leaf area, 100 seed weight and seed yield per plant) were recorded.

### Statistical analysis

The collected data of various aspects of sunflower hybrid were subjected to statistical analysis such as ANOVA, heterosis, heterobeltiosis, and combining ability analysis to understand the yield potential exhibited by each respective sunflower hybrid and to assess the efficacy of heterotic grouping pattern identified. R-Studio version 1.3.1335 was used for statistical analysis of F_1_ cross combinations.

## Results

### Experiment 1

For accurate identification of heterotic grouping pattern, a multi-prong strategy was adopted, wherein morphological, bio-chemical, and molecular datasets of sunflower genotypes were analyzed by using three clustering algorithms, i.e., hierarchical, K-means and hierarchical + K-means hybrid classification algorithm. Efficacy of these three machine learning algorithms were tested on the sunflower genotypes and the algorithm that best explains and accurately classified the genotypes were used for final parental selection for further hybrid development.

#### Hierarchical clustering

**Figure 2:**
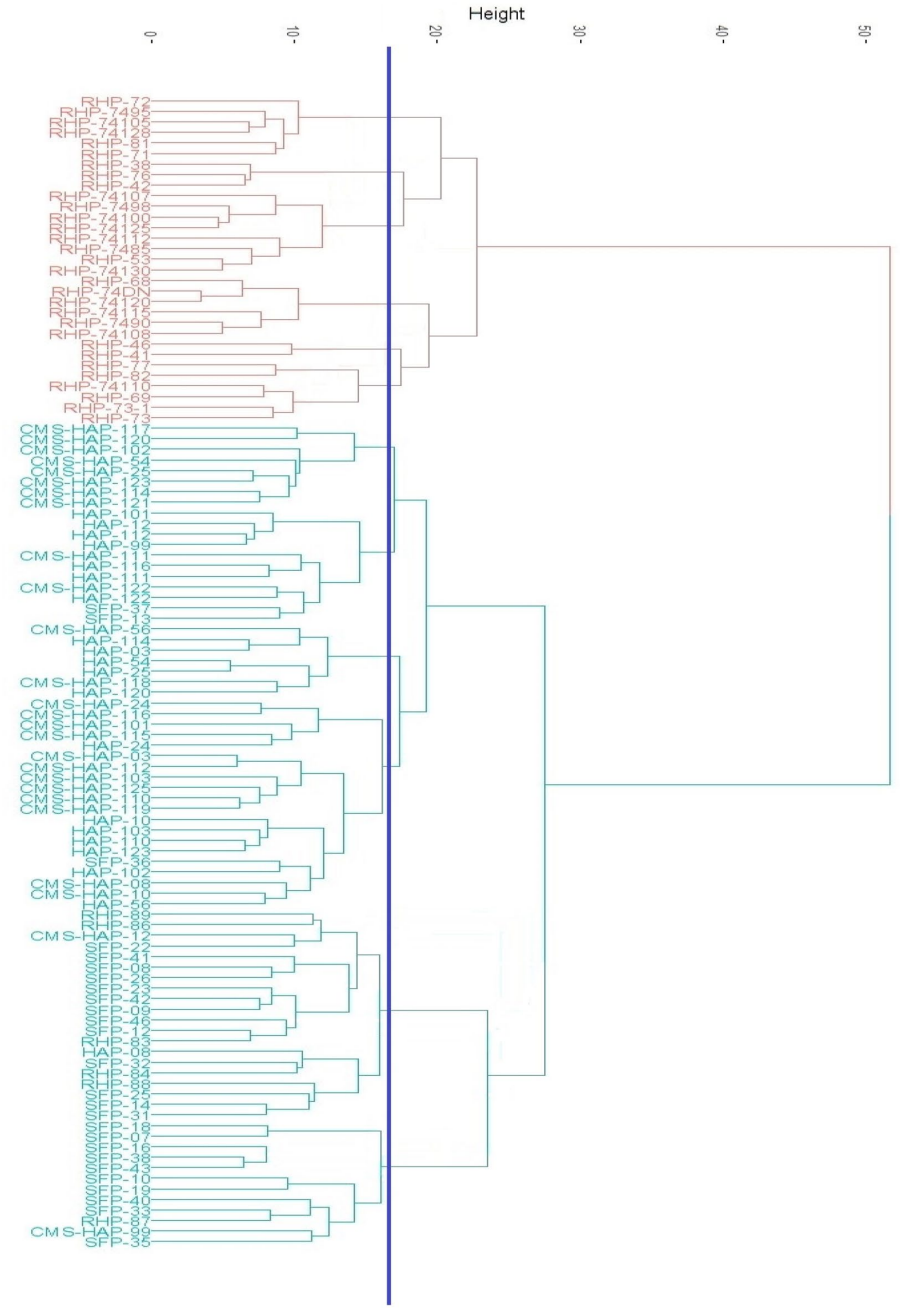
Hierarchical clustering of 109 sunflower genotypes through Ward.D^2^ method.

Figure 2 represents the dendrogram obtained by using hierarchical classification algorithm. For hierarchical clustering, Ward.D^2^ method was applied on combined dataset of morphological + bio-chemical + molecular characterization. Cluster diagram (Fig. 2) showed two distinct classes of genotypes, wherein cluster 1 contains all the restorer lines, while cluster 2 has CMS + B-line and self-pollinated lines. Number of genotypes grouped in cluster 1 includes 31 sunflower genotypes, while the rest 78 sunflower genotypes grouped in cluster 2. Further, at genetic distance of 18, these clusters can be sub-divided into 6 smaller groups. Sub-group 1-A has six genotypes, while there are 3, 8, 6, 2 and 6 genotypes in subgroup 1-B, 1-C, 1-D, 1-E and 1-F respectively. Likewise, Cluster-2 can be divided into six sub-groups at the genetic distance 18. The number of genotypes recorded in sub-group 2-A was 8, while sub-group 2-B had 11 genotypes. Similarly, the number of genotypes recorded in sub-groups 2-C, 2-D, 2-E, and 2-F were 7, 20, 20 and 12 respectively.

#### K-means clustering

**Figure 3:**
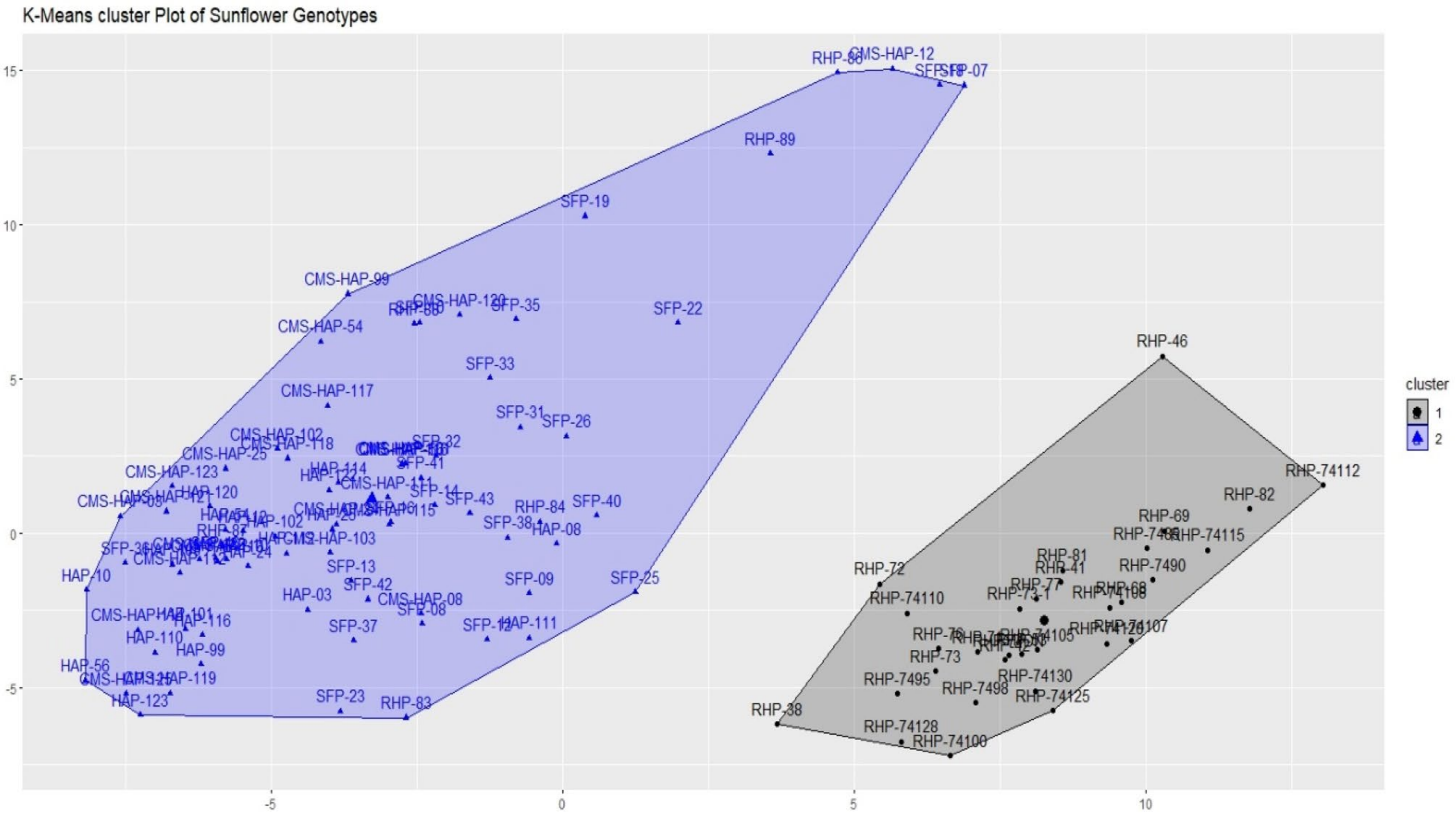
K-means clustering of 109 sunflower genotypes.

K-means cluster algorithm is an unsupervised machine learning based approach that tends to group the similar data points in one cluster, which is away from the dis-matching data points. More precisely, this algorithm aims to minimize the sum of square values within a cluster and consequently maximize the sum of squares between clusters. In the present study, K-means clustering applied on the 109 sunflower genotypes, precisely grouped the sunflower genotypes into 2 major clusters (Fig. 3). The size of cluster 1 is 31, while cluster 2 classified 78 sunflower genotypes. Cluster 1 predominantly contains restorer lines, while cluster 2 contains self-pollinated (SFP) lines i.e. A-lines and B-lines of sunflower genetic pool under study. Although K-means application precisely grouped the sunflower genotypes into two major clusters, selecting genotypes with more precision to smaller groups was not possible using this algorithm. As many SFP lines lie closer to the A-line or B-lines, making it harder to distinguish between them.

#### K-mean, hierarchical hybrid clustering approach

**Figure 4:**
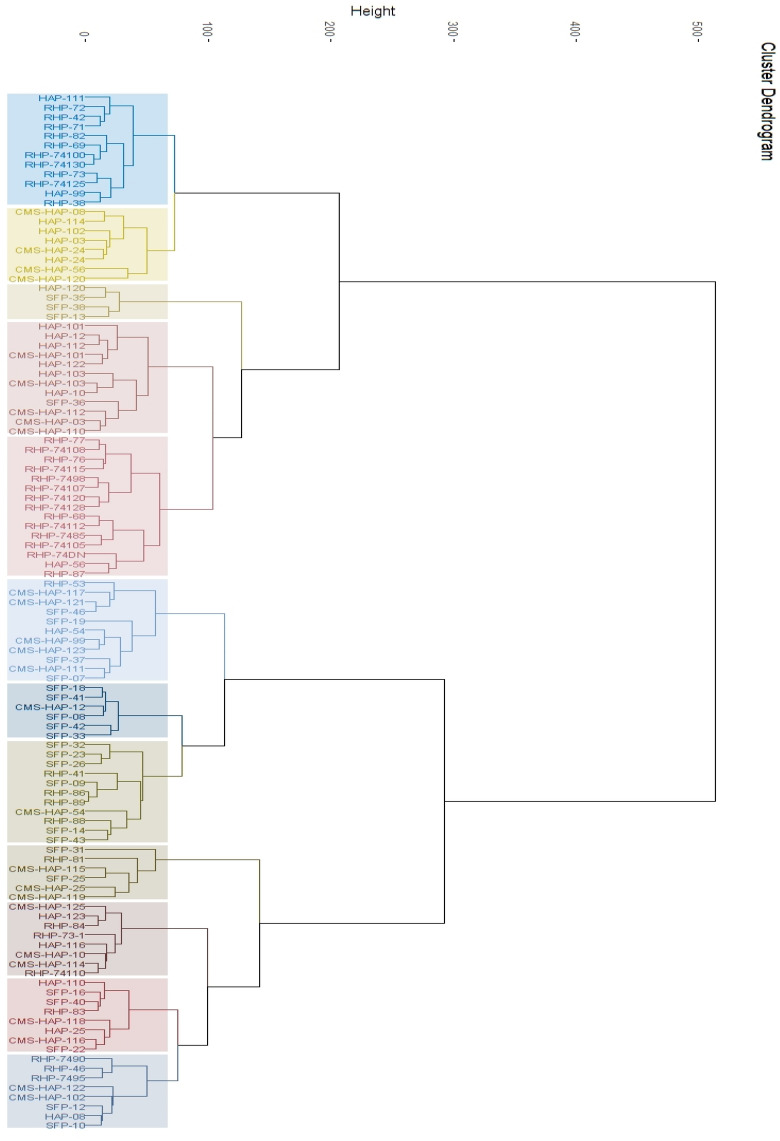
Clustering of 109 sunflower genotypes through hybrid (hierarchical + K-means) machine learning.

Finally, a hybrid algorithm by using hierarchical + K-means clustering algorithms was applied on the sunflower genotypes to examine if the accuracy of harvesting more precise heterotic groups can be improved further or not? Setting the number of k(s) to 12, two major clusters were observed, that were further grouped into 12 smaller clusters (Fig. 4). Cluster 1 contains 12 genotypes in which there were 2 B-lines and 10 restorer lines, cluster 2 contains 8 genotypes (4 CMS + 4 B-lines). Cluster 3 had 4 genotypes (1 B-line + 3 SFP lines), and 12 genotypes (6 CMS-lines, 5 B-lines and 1 SFP line) were grouped into cluster 4. Cluster 5 gathered 15 genotypes which were all Restorer lines, 11 genotypes were grouped in cluster 6 (5 CMS lines, 4 SFP lines, 1 Restorer line and 1 B-line). Likewise, cluster 7 had 6 sunflower genotypes (5 SFP lines + 1 CMS lines), cluster 8 had 11 genotypes (6 SFP lines, 4 restorer lines and 1 CMS line). 6 sunflower genotypes (3 CMS lines, 2 SFP lines and 1 restorer lines) were grouped in cluster 9, while cluster 10 showed a grouping of 8 genotypes (3 CMS lines, 3 Restorer lines and 2 B-lines). Cluster 11 had 8 sunflower genotypes (3 SFP lines, 2 CMS lines, 2 B-lines and 1 Restorer line) and 8 sunflower genotypes tend to group in cluster 12 (3 Restorer, 2 CMS-lines, 2 B-lines and 1 SFP line).

Grouping of sunflower genotypes observed by the application of hybrid algorithm (hierarchical + K-means) was found to be useful to some extent as it can be used to group closer genotypes, however, grouping of genotypes with distinct characteristics like restorer lines and CMS lines closely is somewhat confusing, hence this algorithm is also found to be not a good fit for the current study. As the grouping of genotypes using hierarchical clustering algorithm is clearer and more definitive, hence selection of potential parents for the development of sunflower hybrids were based on the grouping observed through hierarchical clustering approach.

#### Selection of parents

As 12 clusters were observed through hierarchical clustering method, 1 genotype from each of the 12 clusters was selected for further utilization in sunflower hybrid breeding program. Genotypes exhibiting the highest seed yield potential from each of the 12 clusters (recorded at the height of 18) were selected. Moreover, all the restorer lines tend to cluster separately from CMS lines, hence Line × Tester mating design was followed for sunflower hybrid F_1_ development.

### Experiment 2

#### Evaluation of identified heterotic groups

To assess the practical efficiency of the identified heterotic groups, selected parental lines were crossed in Line × Tester mating design and 36 F_1_ hybrids of sunflower were generated. Heterosis (mid-parent heterosis, better parent heterosis) and combining ability analysis (General combining ability and Specific combining ability) were conducted to evaluate the potential of methodology used for identification/mining of heterotic grouping pattern and thereof selection of potential parental lines for commercial hybrid development.

#### Mean performance of parents

Table 1 presents the mean performance of 12 sunflower lines that were planted at NARC, Islamabad. The study focused on nine agro-morphological traits. Among the lines, CMS-HAP-112 exhibited the shortest duration to initiate flowering, taking only 46.5 days, while RHP-41 had the longest duration of 56.5 days. CMS-HAP-111 completed 100% flowering the earliest, within 55 days, followed by CMS-HAP-112 at 55.5 days. On the other hand, RHP-41 took the maximum number of days to complete flowering, with a duration of 67.5 days. Regarding plant height, the 12 parental sunflower lines displayed a range from 200.14 cm (CMS-HAP-54) to 134.6 cm (CMS-HAP-111). In terms of leaf area, CMS-HAP-56 had the highest recorded value of 257.48 cm2, while RHP-38 had the lowest average leaf area of 141.5 cm2. The largest head diameter of 19.3 cm was observed in CMS-HAP-99, whereas the smallest head diameter of 10.45 cm was found in RHP-38. In the context of stem curvature, the lowest value recorded was 6.95 cm for RHP-71, while CMS-HAP-111 and CMS-HAP-12 exhibited the highest stem curvatures of 48 cm and 45.7 cm, respectively. The number of leaves varied among the parental lines, with CMS-HAP-111 having the fewest leaves (23.35), and CMS-HAP-112 having the highest number of leaves (33.1), followed by CMS-HAP-99 (33). The 100 seed weight of the parental lines ranged from 3.48 g (RHP-69) to 6.61 g (CMS-HAP-99). CMS-HAP-112 displayed the highest mean seed yield per plant at 68.19 g, while the lowest seed yield per plant was observed in RHP-68 (27.28 g) and RHP-41 (27.9 g) (Table 1).

**Table 1:** Mean performance of parents regarding morphometric characteristics.

Parents	DFI	DFC	PH	LA	HD	SC	LP	HSW	SYP
CMS-HAP-54	48.5	59	200.14	214.56	17.1	22.05	31.8	5.24	62.83
CMS-HAP-56	48.5	59	188.45	257.48	17.85	40	29.5	5.11	63.29
CMS-HAP-112	46.5	55.5	192.15	197.32	18.15	45.5	33.1	5.11	68.19
CMS-HAP-111	48.5	55	134.6	233.65	17.3	48	23.35	5.65	56.85
CMS-HAP-12	49	57.5	200	171.17	17	45.7	29	5.87	57.05
CMS-HAP-99	49.5	58	186.85	189.8	19.3	26.3	33	6.61	65.05
RHP-68	55	63.5	161.45	167.27	14.55	15.65	25.9	4.26	27.28
RHP-41	56.5	67.5	180.75	220.3	11.05	9.55	30	3.52	27.9
RHP-38	52	63.5	166.5	141.5	10.45	12.95	27.55	4.04	30.29
RHP-53	50.5	60.5	181.4	191.4	13	14.1	32.65	4.04	32.37
RHP-71	55	62	154.35	168.7	12.95	6.95	26.1	3.49	30.8
RHP-69	53.5	61.5	138.55	148.15	11.4	15.7	30.5	3.48	27.45

*DFI* days to flower initiation, *DFC* days to flower completion, *PH* pant height, *LA* leaf area, *HD* head diameter, *S.C* stem curvatures, *LP* leaves per plant, *HSW* 100 seed weight, *SYP* seed yield per plant.

#### Mean performance of hybrids

Table 2 shows the average of 36 sunflower hybrids grown in NARC, Islamabad. The research focused on nine agromorphological traits. Hybrids RHP-68 × CMS-HAP-112 and RHP-38 × CMS-HAP-112 had the shortest flowering times, only 44 days. On the other hand, the hybrid RHP-71 × CMS-HAP-56 had the longest time to flower initiation at 56.5 days. RHP-68 × CMS-HAP-112 and RHP-38 × CMS-HAP-54 showed the minimum number of days (50) required for hybrids to complete 100% flowering, whereas RHP-71 × CMS- HAP-111 was 66 5 days. The number of days until the flowering rate reaches 100%. Regarding the mean leaf area approaching physiological maturity, RHP-71 × CMS-HAP-56 showed the highest value of 176.53 cm^2^, while RHP-69 × CMS-HAP had the lowest mean leaf area. The largest head diameter he recorded with the RHP-71 × CMS-HAP-99 was 23.95 cm, followed by he with the RHP-53 × CMS-HAP-111 with a diameter of 22.77 cm. Conversely, RHP-68 × CMS-HAP-112 had the smallest head diameter of 17.11 cm, followed by RHP-68 × CMS-HAP-54 with 17.53 cm, and the tallest hybrid in terms of plant height was RHP-71 × CMS. -HAP-112 had an average height of 175.17 cm. while the smaller hybrids were RHP-53 × CMS-HAP-111 (131 cm) and RHP-41 × CMS-HAP-56 (132 cm).

**Table 2:** Mean performance of hybrids regarding morphometric characteristics.

Crosses	DFI	DFC	LA	HD	PH	SC	LP	HSW	SY/P
RHP-68 × CMS-HAP-56	45	53	138.77	18.38	146.13	63.38	32.5	5.77	59.24
RHP-68 × CMS-HAP-111	47	55	152.8	19.48	148.08	66.63	31.83	5.6	75.25
RHP-68 × CMS-HAP-112	44	50	161.71	17.11	137.75	59.5	29.33	5.37	50.97
RHP-68 × CMS-HAP-12	49	57	141.2	20.99	152.79	65.42	28.67	6.1	88.93
RHP-68 × CMS-HAP-54	47	55.5	156.03	17.53	129.42	48.83	28.67	6.01	81.5
RHP-68 × CMS-HAP-99	47	53.5	134.89	19.25	159.33	77.5	30.17	5.8	72.32
RHP-41 × CMS-HAP-111	50	56	145.15	20.18	157.8	66.79	33	6.43	99.45
RHP-41 × CMS-HAP-12	50	56	145.6	20.16	137	61.67	27.67	6.38	87.93
RHP-41 × CMS-HAP-54	47.5	53.5	174.44	19.33	145.67	58.33	28.17	5.77	61
RHP-41 × CMS-HAP-56	46	52.5	133.17	19.79	132	56.83	29	6.07	68.12
RHP-41 × CMS-HAP-112	45	51.5	129.65	19.99	141	59.67	29.33	6.03	73.21
RHP-41 × CMS-HAP-99	49	57.5	166.2	21.18	157.5	63	34	6.59	92.98
RHP-38 × CMS-HAP-111	48.5	56.5	152.23	19.55	171.5	64.33	31.67	4.77	60.63
RHP-38 × CMS-HAP-56	45	51	142.55	18.14	153.5	74.83	29.83	6.26	70.92
RHP-38 × CMS-HAP-112	44	50.5	148.44	17.52	145.5	69.33	32.67	6.55	64.74
RHP-38 × CMS-HAP-54	44.5	50	178.5	19.65	139.33	64.83	33.17	7.07	72.66
RHP-38 × CMS-HAP-12	49.5	55.5	162.01	21.89	159.17	57.67	33.83	7.34	74.82
RHP-38 × CMS-HAP-99	47.5	55	155.7	21.25	155.5	55	33.33	6.63	79.6
RHP-53 × CMS-HAP-111	49.5	56	160.07	22.77	131	57.83	26	4.94	49.3
RHP-53 × CMS-HAP-54	45	51	161.92	18.95	130.88	51.17	27	6.17	59.53
RHP-53 × CMS-HAP-12	49	55.5	165	19.38	137	60.33	32.33	5.48	57.92
RHP-53 × CMS-HAP-112	45	51.5	162.62	18.95	135.17	57.33	29.83	5.63	49.38
RHP-53 × CMS-HAP-99	47.5	55	165.02	19.07	144.83	66.33	33.33	6.3	65.26
RHP-53 × CMS-HAP-56	45	51	146.57	19.57	149	62.5	29	7.01	61.88
RHP-71 × CMS-HAP-99	56	64	173.29	23.95	155	60.5	36.17	5.6	92.89
RHP-71 × CMS-HAP-56	56.5	65.5	176.53	21.89	161.33	55.33	36.67	5.71	92.24
RHP-71 × CMS-HAP-112	55	64	158.77	22.54	175.17	55	36.33	5.21	87.77
RHP-71 × CMS-HAP-111	57	66.5	169.77	22.61	162.33	58.83	35.5	4.41	90.93
RHP-71 × CMS-HAP-54	56	64	169.14	21.79	164.17	60.5	36	5.1	103.36
RHP-71 × CMS-HAP-12	57	64	163.07	22.39	151	55.17	35.67	5.57	98.91
RHP-69 × CMS-HAP-12	49	54.5	146.4	20.98	156.5	59.83	29.5	6.23	73.57
RHP-69 × CMS-HAP-112	45	51.5	132.38	17.81	141.33	57.5	30.67	5.9	60.71
RHP-69 × CMS-HAP-111	49.5	54.5	144.71	19.82	137	52.83	33.5	5.57	70.66
RHP-69 × CMS-HAP-56	46	51.5	143.27	18.5	141.33	57.5	29.83	6.77	66.72
RHP-69 × CMS-HAP-54	46.5	52	126.04	18.01	146.5	55	31.83	6.73	74.13
RHP-69 × CMS-HAP-99	49.5	54	149.43	18.01	149.54	70.04	29	6.76	71.67

*DFI* days to flower initiation, *DFC* days to flower completion, *PH* pant height, *LA* leaf area, *HD* head diameter, *S.C* stem curvatures, *LP* leaves per plant, *HSW* 100 seed weight, *SYP* seed yield per plant.

Regarding stem curvature, the lowest recorded value was 42.77 cm for RHP-68 × CMS-HAP-54, followed by RHP-53 × CMS-HAP-54 with a stem curvature of 48.83 cm. HAP-99 and RHP-38 × CMS-HAP-112 exhibited maximum stem curvatures of 77.5 cm and 74.83 cm, respectively. RHP-53 × CMS-HAP-111 has the lowest number of seats (26), RHP-71 × CMS-HAP-56 has the highest number of seats (36.67), followed by RHP-71 × CMS-HAP-99 continued. (36.17). Test weights of hybrids ranged from 4.41 g (RHP-71 × CMS-HAP-111) to 7.34 g (RHP-38 × CMS-HAP-12). The minimum seed yield per plant for hybrid RHP-53 × CMS-HAP-111 was 49.3 g, whereas RHP-71 × CMS-HAP-54 showed the highest average seed yield of 103.36 g per plant, compared to RHP-41 followed by RHP-41 × CMS-HAP-111 of 99.45 g.

#### Heterosis and heterobeltiosis

Results of heterosis and heterobeltiosis for nine morphological characteristics of sunflower plants are presented in Table 3 and 4. Range of heterosis for days to flower initiation reported in present study was from 10.14**% (CMS-HAP-111 × RHP-71) to − 13.04% (CMS-HAP-56 × RHP-68). The heterotic effects of six hybrids were found to be in positive direction, while non-significant heterosis effects were found of six cross combinations. Remaining all cross combinations showed a highly significant heterosis for days to flower initiation. Heterobeltiotic effects recorded for 36 sunflower hybrids were found to be in the range of − 20.35% (CMS-HAP-112 × RHP-41) to 3.65*% (CMS-HAP-111 × RHP-71). Most of heterobeltiotic effects are in negative direction.

**Table 3:** Heterosis (mid parent) and heterobeltiosis (better parent) of 36 sunflower hybrids.

Crosses/hybrids	DFI		DFC		LA		HD		PH	
	MPH	BPH	MPH	BPH	MPH	BPH	MPH	BPH	MPH	BPH
CMS-HAP-56 × RHP-68	− 13.04**	− 18.18**	− 13.47**	− 16.54**	− 34.66**	− 46.11**	13.43*	2.94 ns	− 16.48**	− 22.46**
CMS-HAP-56 × RHP-41	− 12.38**	− 18.58**	− 17**	− 22.22**	− 44.26**	− 48.28**	36.96**	10.87 ns	− 28.49**	− 29.95**
CMS-HAP-56 × RHP-38	− 10.45**	− 13.46**	− 16.73**	− 19.69**	− 28.54**	− 44.64**	28.2**	1.62 ns	− 13.51**	− 18.55**
CMS-HAP-56 × RHP-53	− 9.09**	− 10.89**	− 14.64**	− 15.7**	− 34.7**	− 43.08**	26.9**	9.66 ns	− 19.43**	− 20.93**
CMS-HAP-56 × RHP-71	9.18**	2.73 ns	8.26**	5.65*	− 17.16**	− 31.44**	42.14**	22.63**	− 5.87 ns	− 14.39*
CMS-HAP-56 × RHP-69	− 9.8**	− 14.02**	− 14.52**	− 16.26**	− 29.36**	− 44.36**	26.5**	3.64 ns	− 13.56*	− 25**
CMS-HAP-111 × RHP-68	− 9.18**	− 14.55**	− 7.17**	− 13.39**	− 23.78**	− 34.6**	22.32**	12.6 ns	0.04 ns	− 8.28 ns
CMS-HAP-111 × RHP-41	− 4.76*	− 11.5**	− 8.57**	− 17.04**	− 36.05**	− 37.88**	42.4**	16.68*	0.08 ns	− 12.7*
CMS-HAP-111 × RHP-38	− 3.48 ns	− 6.73**	− 4.64*	− 11.02**	− 18.84*	− 34.85**	40.94**	13.03 ns	13.92*	3 ns
CMS-HAP-111 × RHP-53	0 ns	− 1.98 ns	− 3.03 ns	− 7.44**	− 24.68**	− 31.5**	50.33**	31.65**	− 17.09**	− 27.78**
CMS-HAP-111 × RHP-71	10.14**	3.65*	13.68**	7.26**	− 15.61*	− 27.34**	49.52**	30.72**	12.36 ns	5.17*
CMS-HAP-111 × RHP-69	− 2.94 ns	− 7.48**	− 6.44**	− 11.38**	− 24.19**	− 38.06**	38.08**	14.54*	0.31 ns	− 1.12 ns
CMS-HAP-112 × RHP-68	− 13.3**	− 20**	− 15.97**	− 21.26**	− 11.29 ns	− 18.04*	4.65 ns	− 5.73 ns	− 22.09**	− 28.31**
CMS-HAP-112 × RHP-41	− 12.62**	− 20.35**	− 16.26**	− 23.7**	− 37.91**	− 41.15**	36.92**	10.14 ns	− 24.38**	− 26.62**
CMS-HAP-112 × RHP-38	− 10.66**	− 15.38**	− 15.13**	− 20.47**	− 12.37 ns	− 24.77**	22.55**	− 3.44 ns	− 18.86**	− 24.28**
CMS-HAP-112 × RHP-53	− 7.22**	− 10.89**	− 11.21**	− 14.88**	− 16.33*	− 17.59*	21.67**	4.41 ns	− 27.63**	− 29.66**
CMS-HAP-112 × RHP-71	8.37**	0 ns	8.94**	3.23 ns	− 13.25 ns	− 19.54*	44.95**	24.19**	1.11 ns	− 8.84 ns
CMS-HAP-112 × RHP-69	− 10**	− 15.89**	− 11.97**	− 16.26**	− 23.36**	− 32.91**	20.58**	− 1.85 ns	− 14.52**	− 26.45**
CMS-HAP-12 × RHP-68	− 5.77**	− 10.91**	− 5.79**	− 10.24**	− 16.56*	− 17.51 ns	33.09**	23.5**	− 15.46**	− 23.6**
CMS-HAP-12 × RHP-41	− 5.21*	− 11.5**	− 10.4**	− 17.04**	− 25.61**	− 33.91**	43.78**	18.62*	− 28.04**	− 31.5**
CMS-HAP-12 × RHP-38	− 1.98 ns	− 4.81*	− 8.26**	− 12.6**	3.63 ns	− 5.35 ns	59.49**	28.76**	− 13.14**	− 20.42**
CMS-HAP-12 × RHP-53	− 1.51 ns	− 2.97 ns	− 5.93**	− 8.26**	− 8.98 ns	− 13.79 ns	29.17**	13.97 ns	− 28.16**	− 31.5**
CMS-HAP-12 × RHP-71	9.62**	3.64*	7.11**	3.23 ns	− 4.04 ns	− 4.74 ns	49.52**	31.71**	− 14.77**	− 24.5**
CMS-HAP-12 × RHP-69	− 4.39*	− 8.41**	− 8.4**	− 11.38**	− 8.31 ns	− 14.47 ns	47.78**	23.44**	− 7.55 ns	− 21.75**
CMS-HAP-54 × RHP-68	− 9.18**	− 14.55**	− 9.39**	− 12.6**	− 18.27*	− 27.28**	10.74 ns	2.49 ns	− 28.42**	− 35.34**
CMS-HAP-54 × RHP-41	− 9.52**	− 15.93**	− 15.42**	− 20.74**	− 19.77**	− 20.82**	37.3**	13.01 ns	− 23.51**	− 27.22**
CMS-HAP-54 × RHP-38	− 11.44**	− 14.42**	− 18.37**	− 21.26**	0.26 ns	− 16.81*	42.69**	14.94*	− 24**	− 30.38**
CMS-HAP-54 × RHP-53	− 9.09**	− 10.89**	− 14.64**	− 15.7**	− 20.23**	− 24.54**	25.91**	10.82 ns	− 31.4**	− 34.61**
CMS-HAP-54 × RHP-71	8.21**	1.82 ns	5.79**	3.23 ns	− 11.74 ns	− 21.17**	45.02**	27.43**	− 7.38 ns	− 17.97**
CMS-HAP-54 × RHP-69	− 8.82**	− 13.08**	− 13.69**	− 15.45**	− 30.5**	− 41.26**	26.39**	5.32 ns	− 13.49*	− 26.8**
CMS-HAP-99 × RHP-68	− 10.05**	− 14.55**	− 11.93**	− 15.75**	− 24.44**	− 28.93**	13.74*	− 0.26 ns	− 8.51 ns	− 14.73*
CMS-HAP-99 × RHP-41	− 7.55**	− 13.27**	− 8.37**	− 14.81**	− 18.95**	− 24.56**	39.54**	9.72 ns	− 14.31**	− 15.71**
CMS-HAP-99 × RHP-38	− 6.4**	− 8.65**	− 9.47**	− 13.39**	− 6.01 ns	− 17.97*	42.89**	10.13 ns	− 11.99*	− 16.78**
CMS-HAP-99 × RHP-53	− 5*	− 5.94*	− 7.17**	− 9.09**	− 13.42 ns	− 13.78 ns	18.11**	− 1.17 ns	− 21.34**	− 22.49**
CMS-HAP-99 × RHP-71	7.18**	1.82 ns	6.67**	3.23 ns	− 3.32 ns	− 8.7 ns	48.56**	24.12**	− 9.14 ns	− 17.05**
CMS-HAP-99 × RHP-69	− 3.88 ns	− 7.48**	− 9.62**	− 12.2**	− 11.57 ns	− 21.27*	17.33*	− 6.68 ns	− 8.09 ns	− 19.97**

*MPH* mid-parent heterosis, *BPH* better parent heterosis, *significant, **highly significant, *ns* non-significant, *DFI* days to flower initiation, *DFC* days to flower completion, *LA* leaf area, *HD* head diameter, *PH* plant height.

**Table 4:** Heterosis (mid parent) and heterobeltiosis (better parent) of 36 sunflower hybrids.

Crosses/hybrids	SC		LP		HSW		SYP	
	MPH	BPH	MPH	BPH	MPH	BPH	MPH	BPH
CMS-HAP-56 × RHP-68	127.76**	58.44**	17.33**	10.17 ns	23.22*	12.92 ns	30.82 ns	− 6.39 ns
CMS-HAP-56 × RHP-41	129.4**	42.09*	− 2.52 ns	− 3.33 ns	40.59**	18.79 ns	49.4*	7.63 ns
CMS-HAP-56 × RHP-38	182.66**	87.09**	4.59 ns	1.14 ns	36.65**	22.41*	51.58*	12.06 ns
CMS-HAP-56 × RHP-53	131.05**	56.25**	− 6.68 ns	− 11.18 ns	53.14**	37.18**	29.37 ns	− 2.23 ns
CMS-HAP-56 × RHP-71	135.72**	38.34*	31.89**	24.29**	32.75**	11.64 ns	96.08**	45.75*
CMS-HAP-56 × RHP-69	106.46**	43.75*	− 0.55 ns	− 2.18 ns	57.72**	32.49**	47.07*	5.43 ns
CMS-HAP-111 × RHP-68	109.35**	38.8*	29.26**	22.9**	13.07 ns	− 0.88 ns	78.89**	32.37 ns
CMS-HAP-111 × RHP-41	132.13**	39.16*	23.71**	10 ns	40.16**	13.81 ns	134.69**	74.93**
CMS-HAP-111 × RHP-38	111.11**	34.03*	24.44**	14.95*	− 1.5 ns	− 15.49*	39.16 ns	6.65 ns
CMS-HAP-111 × RHP-53	86.25**	20.48 ns	− 7.14 ns	− 20.37**	2.01 ns	− 12.48 ns	10.52 ns	− 13.27 ns
CMS-HAP-111 × RHP-71	114.12**	22.56 ns	43.58**	36.02**	− 3.45 ns	− 21.95*	107.48**	59.95**
CMS-HAP-111 × RHP-69	65.87**	10.06 ns	24.42**	9.84 ns	22.08*	− 1.42 ns	67.64**	24.29 ns
CMS-HAP-112 × RHP-68	94.6**	30.77 ns	− 0.56 ns	− 11.37 ns	14.79 ns	5.19 ns	6.78 ns	− 25.25 ns
CMS-HAP-112 × RHP-41	116.77**	31.13 ns	− 7.04 ns	− 11.39 ns	39.55**	17.91 ns	52.4*	7.38 ns
CMS-HAP-112 × RHP-38	137.25**	52.38**	7.73 ns	− 1.3 ns	43.09**	28.18*	31.48 ns	− 5.06 ns
CMS-HAP-112 × RHP-53	92.4**	26.01 ns	− 9.25 ns	− 9.86 ns	22.88*	10.08 ns	− 1.79 ns	− 27.58 ns
CMS-HAP-112 × RHP-71	109.72**	20.88 ns	22.74**	9.76 ns	21.12 ns	1.86 ns	77.34**	28.72 ns
CMS-HAP-112 × RHP-69	87.91**	26.37 ns	− 3.55 ns	− 7.34 ns	37.33**	15.36 ns	26.97 ns	− 10.96 ns
CMS-HAP-12 × RHP-68	113.25**	43.14**	4.43 ns	− 1.16 ns	20.45*	3.92 ns	110.89**	55.87*
CMS-HAP-12 × RHP-41	123.22**	34.93*	− 6.2 ns	− 7.77 ns	35.89**	8.78 ns	107.02**	54.13*
CMS-HAP-12 × RHP-38	96.64**	26.18 ns	19.65**	16.66*	48.03**	25.06*	71.35**	31.16 ns
CMS-HAP-12 × RHP-53	101.79**	32.02 ns	4.88 ns	− 0.98 ns	10.6 ns	− 6.56 ns	29.56 ns	1.53 ns
CMS-HAP-12 × RHP-71	109.55**	20.71 ns	29.46**	22.98**	19.25 ns	− 4.94 ns	125.18**	73.37**
CMS-HAP-12 × RHP-69	94.9**	30.93 ns	− 0.84 ns	− 3.28 ns	33.4**	6.22 ns	74.13**	28.96 ns
CMS-HAP-54 × RHP-68	159.07**	121.47**	− 0.64 ns	− 9.86 ns	26.53*	14.59 ns	80.89**	29.73 ns
CMS-HAP-54 × RHP-41	269.21**	164.56**	− 8.85 ns	− 11.43 ns	31.7**	10.1 ns	34.47 ns	− 2.9 ns
CMS-HAP-54 × RHP-38	270.46**	194.01**	11.76*	4.29 ns	52.21**	34.8**	56.06*	15.65 ns
CMS-HAP-54 × RHP-53	183.1**	132.06**	− 16.21**	− 17.3**	32.83**	17.64	25.06 ns	− 5.25 ns
CMS-HAP-54 × RHP-71	317.24**	174.38**	24.35**	13.21*	16.84 ns	− 2.76 ns	120.81**	64.53**
CMS-HAP-54 × RHP-69	191.39**	149.43**	2.2 ns	0.11 ns	54.24**	28.22*	64.24**	18 ns
CMS-HAP-99 × RHP-68	269.49**	194.68**	2.44 ns	− 8.58 ns	6.81 ns	− 12.19 ns	56.66*	11.18 ns
CMS-HAP-99 × RHP-41	251.46**	139.54**	7.94 ns	3.03 ns	30.11**	− 0.23 ns	100.05**	42.93*
CMS-HAP-99 × RHP-38	180.25**	109.13**	10.11 ns	1.02 ns	24.6**	0.45 ns	67**	22.38 ns
CMS-HAP-99 × RHP-53	228.39**	152.22**	1.55 ns	1.02 ns	18.4*	− 4.54 ns	33.99 ns	0.33 ns
CMS-HAP-99 × RHP-71	263.91**	130.04**	22.39**	9.59 ns	11 ns	− 15.22 ns	93.82**	42.8*
CMS-HAP-99 × RHP-69	233.52**	166.31**	− 8.66 ns	− 12.12*	34.03**	2.27 ns	54.96*	10.18 ns

*MPH* mid-parent heterosis, *BPH* better parent heterosis, *significant, **highly significant, *ns* non-significant, *SC* stem curvatures, *LP* leaves per plant, *HSW* 100 seed weight, *SYP* seed yield per plant.

CMS-HAP-54 × RHP-38 showed the maximum heterotic effect in negative direction for days taken to 100% flowering (− 18.37**%) followed by CMS-HAP-56 × RHP-41 (− 17.0**%) and CMS-HAP-56 xRHP-38 (− 16.73**%). Whereas hybrid CMS-HAP-111 × RHP-71 depicted the highest positive heterotic effect for this trait (13.68**%) followed by CMS-HAP-12 × RHP-71 (8.94**%). The heterotic effect was significant for all hybrids except for CMS-HAP-111 × RHP-53. Range of heterobeltiosis was recorded from -23.7**% (CMS-HAP-112 × RHP-41) to 7.26**% (CMS-HAP-111 × RHP-71). Heterobeltiotic effect of all the hybrid combinations found to be statistically highly significant for days to complete flowering except four hybrids viz., CMS-HAP-112 × RHP-71, CMS-HAP-12 × RHP-71, CMS-HAP-54 × RHP-71 and CMS-HAP-99 × RHP-71.

Results obtained of heterosis and heterobeltiosis effects for leaf area in hybrid combination under study depicted that heterosis over mid parent ranged from 3.63^ns^% to − 44.26**%. Highest magnitude of positive heterosis effect was noted for CMS-HAP-12 × RHP-38 (3.63^ns^%) while negative heterotic effect in negative direction was recorded for F_1_ hybrid CMS-HAP-56 × RHP-41 (− 44.26**%). Highest effect for heterobeltiosis observed in negative direction was (− 48.28**%) for CMS-HAP-56 × RHP-41, followed by CMS-HAP-56 × RHP-68 (− 46.11**%). Heterobeltiotic effects of 29 hybrids was found to be statistically significant.

Maximum heterosis for head diameter was observed for CMS-HAP-12 × RHP-38 (59.49**%), whereas lowest magnitude of mid parent heterosis was depicted by CMS-HAP-112 × RHP-68(4.65^ns^%) (Table 3). All hybrids exhibited positive mid parent heterosis. Maximum heterobeltiosis was observed for CMS-HAP-12 × RHP-71 (31.71**%), while minimum heterobeltiosis was recorded for CMS-HAP-99 × RHP-69 (− 6.68^ ns^%). Only six sunflower hybrids showed a negative heterobeltiotic effect for head diameter. Maximum mid parent heterosis for plant height recorded was − 31.4**% (CMS-HAP-54 × RHP-53), while minimum mid parent heterosis of 13.92*% was observed for CMS-HAP-111 × RHP-38. As many as thirty hybrids exhibited a negative magnitude of mid parent heterosis for head diameter in the present study. Range of heterobeltiosis observed was from − 35.34% (CMS-HAP-54 × RHP-68) to 5.17*% (CMS-HAP-111 × RHP-71). Results for heterobeltiosis of 34 hybrids were found to be negative with respect to better parent heterosis.

Range of heterotic effects for the 36 sunflower hybrids under study recorded was from 65.87**% (CMS-HAP-111 × RHP-69) to 317.24**% (CMS-HAP-54 × RHP-71). All sunflower F_1_ hybrid combinations under study expressed highly significant positive heterotic effects for stem curvature. Heterobeltiosis was statistically significant for 24 hybrids and all 36 F_1_ hybrids showed positive heterotic effects over the best parent. Maximum heterobeltiosis observed was for CMS-HAP-99 × RHP-68 (194.68**%), while minimum heterobeltiosis was recorded for CMS-HAP-111 × RHP-69 (10.06^ns^%). Results for number of leaves per plant obtained depicted that maximum positive heterosis was recorded for CMS-HAP-111 × RHP-71 (45.58**%) followed by CMS-HAP-56 × RHP-71 (31.89**%). Maximum magnitude of negative heterotic effect was noted for CMS-HAP-112 × RHP-53 (− 9.25^ns^%), followed by CMS-HAP-99 × RHP-69 (− 8.66^ns^%). Of all the 36 hybrid combinations under study, 22 expressed positive heterosis for the average number of leaves per plant. Highest magnitude of heterobeltiotic effect in negative direction was recorded for CMS-HAP-111 × RHP-53 (− 20.37**%) while maximum better parent positive heterosis was noted for CMS-HAP-111 × RHP-71 (36.02**%) followed by CMS-HAP-56 × RHP-71 (24.29**%).

Among all the hybrids tested the results of 25 hybrids for 100 seed weight was found to be statistically significant (Table 4). Maximum heterotic effect noted for this character was 57.72**% (CMS-HAP-56 × RHP-69) while minimum mid-parent heterosis observed was − 3.45^ns^% (CMS-HAP-111 × RHP-71). Only two hybrid combinations expressed heterosis for 100 seed weight in negative direction. Heterosis over better parent for 100 seed weight ranges from − 15.49*% (CMS-HAP-111 × RHP-38) to 37.18**% (CMS-HAP-56 × RHP-53). Results of 10 hybrid combinations were found to be statistically significant. Heterobeltiotic effect of 24 hybrids were on positive side (Table 4). Among all the 36 hybrids tested, 35 sunflower hybrids expressed a positive mid parent heterosis for seed yield per plant. The maximum heterotic effect noted for this character was 134.69**% (CMS-HAP-111 × RHP-41) followed by 125.18**% (CMS-HAP-12 × RHP-71) and minimum mid-parent heterosis observed was − 1.79^ns^ (CMS-HAP-112 × RHP-53). Maximum heterobeltiosis recorded was 74.93**% (CMS-HAP-11 × RHP-41) while minimum heterobeltiosis noted was − 27.58^ns^% (CMS-HAP-112 × RHP-53). Heterobeltiotic effect of only nine hybrids were negative while rest of 27 hybrids expressed a positive gain over their better parent for seed yield per plant (Table 4).

#### Combining ability analysis

Line × Tester mating design had the ability to evaluate a greater number of hybrids than the diallel and partial diallel mating designs. This technique of hybrid evaluation is quite successful in cases where hybrids must be developed from Restorer and complete male sterile lines. Results pertaining to General Combining Ability of 12 parental lines are presented in Table 5.

**Table 5:** Estimates of general combining ability (GCA) of parents regarding morphometric characteristics.

	DFI	DFC	LA	HD	PH	SC	LP	HSW	SYP
LINES									
CMS-HAP-54	− 2.1**	− 1.43**	− 6.13 ns	− 1.17**	− 2.7 ns	2.79 ns	− 1.33*	− 0.21 ns	− 2.55 ns
CMS-HAP-56	− 0.68*	− 0.93*	− 4.66 ns	0.15 ns	− 3.12 ns	0.3 ns	− 1.33*	0.22 ns	6.53 ns
CMS-HAP-112	− 2.1**	− 2.35**	2.88 ns	− 0.29 ns	5.8 ns	3.58 ns	0.89 ns	0.45*	− 3.36 ns
CMS-HAP-111	− 1.76**	− 2.1**	6.5 ns	− 0.17 ns	− 10.3**	− 1.5 ns	− 1.94**	− 0.07Ns	− 16.71**
CMS-HAP-12	7.65**	9.24**	14.73**	2.57**	13.22**	− 3.2 ns	4.53**	− 0.72**	20.43**
CMS-HAP-99	− 1.01**	− 2.43**	− 13.32**	− 1.1**	− 2.91 ns	− 1.97 ns	− 0.81 ns	0.34 ns	− 4.34 ns
TESTERS									
RHP-68	1.07**	1.24**	− 1.04 ns	1.02*	4.71 ns	1.36 ns	− 0.06 ns	− 0.37*	− 1.41 ns
RHP-41	− 0.51 ns	− 0.43 ns	− 1.73 ns	− 0.55 ns	− 2.92 ns	0.44Ns	− 0.92 ns	0.01 ns	0.51 ns
RHP-38	− 0.43 ns	− 0.76 ns	5.15 ns	− 0.67 ns	− 1.93 ns	− 1.53 ns	0.53 ns	− 0.33 ns	− 8.41*
RHP-53	− 0.68*	− 0.6 ns	1.06 ns	0.13 ns	− 4.45 ns	− 0.63 ns	− 0.53 ns	0.02 ns	− 1.13 ns
RHP-71	− 0.01 ns	0.15 ns	− 2.38 ns	− 0.24 ns	− 0.77 ns	− 2.75 ns	0.64 ns	0.26Ns	4.8 ns
RHP-69	0.57 ns	0.4 ns	− 1.05 ns	0.32 ns	5.37 ns	3.12 ns	0.33 ns	0.41*	5.64 ns

*DFI* days to flower initiation, *DFC* days to flower completion, *PH* pant height, *LA* leaf area, *HD* head diameter, *S.C* stem curvatures, *LP* Leaves per plant, *HSW* 100 seed weight, *SYP* seed yield per plant.

#### General combining ability (GCA)

Pursual of GCA estimates of all 12 hybrids for DFI showed that only two parents, one CMS, i.e., CMS-HAP-12 (7.65**) and one R-line i.e., RHP-68 (1.07**) had positive and significant GCA effects. Similarly, the same two parents had the highest, positive and significant GCA effect for DFC, depicting that these hybrids are late maturing. For leaf area GCA estimates, CMS-HAP-12 (14.73**) were found to be highly significant and positive among all the 12 parental lines under examination, while CMS-HAP-99 showed the lowest GCA magnitude of − 13.99**. GCA effects for average leaf area for all the six male lines were found to be non-significant. Range of GCA estimates for head diameter recorded was from 2.57** (CMS-HAP-12) to − 1.17** (CMS-HAP-54), while among male lines RHP-68 was found to be a good general combiner for head diameter with GCA effect of 1.02*. The best general combining ability recorded for plant height was from CMS-HAP-12 (13.22**), while lowest GCA estimate of − 10.3** was shown by CMS-HAP-111. Stem curvature GCA estimates of all the 12 parents under study were found to be statistically non-significant. GCA of number of leaves per plant were highly significant for two CMS lines viz., CMS-HAP-111 (− 1.94**) and CMS-HAP-12 (4.53**). RHP-71 (0.64^ns^) showed the maximum GCA among tester lines. For 100 seed weight only 2 parental lines i.e., CMS-HAP-112 (0.45*) and RHP-69 (0.41*) showed good general combining ability for this yield related important plant characteristic. CMS-HAP-12 exhibited highest GCA effect of 20.43** for seed yield per plant among female lines, while for testers no male line exhibited a significant positive GCA effect for seed yield.

#### Specific combining ability (SCA)

Result of combination specific combining ability of thirty-six sunflower hybrids developed from 12 parental line following L × T mating design for nine agro-morphological traits are presented in Table 6. SCA effect of CMS-HAP-12 × RHP-68 (3.18**) was the highest for DFI, while SCA estimate of − 2.9** showed by CMS-HAP-112 × RHP-41 was the lowest in magnitude. Combination specific combining ability estimates for days taken to flower completion was found to be highest for CMS-HAP-12 × RHP-68 (3.60**), while CMS-HAP-112 × RHP-68 cross combination recorded maximum negative SCA effect for DFC, showing that this cross combination is the earliest in flowering than rest of hybrids study. Significant SCA estimates were recorded for all the 36 hybrids for leaf area with maximum SCA effect of 20.87** was observed for CMS-HAP-54 × RHP-38. Only three hybrids showed a positive and significant SCA magnitude for head diameter, with maximum value of 2.46* (CMS-HAP-12 × RHP-38). For head diameter, 21 hybrid combination depicted a negative SCA estimates showing that head diameter of hybrids was less than that of their respective parents. The highest magnitude of SCA for plant height was shown by CMS-HAP-112 × RHP-71 (15.6*). Combination specific combining ability estimates for stem curvature were positive for 34 cross combinations. Range of SCA effects for number of leaves per plant was from 3.47* (CMS-HAP-99 × RHP-41) to − 3.53* (CMS-HAP-11 × RHP-53). Only one cross combination was found to be significant for head diameter SCA effect and in negative direction, i.e., CMS-HAP-111 × RHP-38 (− 1.30**). Positive SCA effects of 17 hybrids for 100 seed weight was observed. For seed yield per plant magnitude of SCA recorded was positive for 19 cross combinations, while maximum positive SCA magnitude was depicted by CMS-HAP-111 × RHP-53 (3.60**) followed by CMS-HAP-112 × RHP-53 (2.93**).

**Table 6:** Estimates of specific combining ability (SCA) of 36 sunflower hybrids regarding morphometric characteristics.

	DFI	DFC	LA	HD	PH	SC	LP	HSW	SYP
CMS-HAP-56 × RHP-68	− 2.57**	− 2.24*	− 7.76 ns	− 1.43 ns	− 4.17 ns	− 1.53 ns	2.36 ns	0.36 ns	− 2.24*
CMS-HAP-56 × RHP-41	− 1.24 ns	− 1.4 ns	− 16.93 ns	− 0.44 ns	− 8.71 ns	− 3.59 ns	− 0.67 ns	− 0.16 ns	1.43 ns
CMS-HAP-56 × RHP-38	− 0.99 ns	− 1.65 ns	− 12.29 ns	− 0.98 ns	2.34 ns	10.06 ns	− 1.67 ns	− 0.19 ns	− 3.15**
CMS-HAP-56 × RHP-53	− 2.4**	− 2.74**	− 12.58 ns	− 0.53 ns	5.65 ns	0.13 ns	− 0.92 ns	0.69 ns	0.26 ns
CMS-HAP-56 × RHP-71	0.76 ns	1.26 ns	9.84 ns	− 0.09 ns	2.76 ns	− 2.66 ns	1.53 ns	0.43 ns	− 0.24 ns
CMS-HAP-56 × RHP-69	− 0.9 ns	− 0.9 ns	1.84 ns	− 0.48 ns	0.42 ns	− 0.66 ns	− 0.36 ns	0.43 ns	1.51 ns
CMS-HAP-111 × RHP-68	1.01 ns	1.43 ns	6.96 ns	1.24 ns	5.43 ns	2.65 ns	2.56 ns	− 0.19 ns	− 1.4 ns
CMS-HAP-111 × RHP-41	1.01 ns	0.26 ns	− 2.84 ns	− 0.94 ns	7.93 ns	4.39 ns	2.86 ns	0.58 ns	1.43 ns
CMS-HAP-111 × RHP-38	0.93 ns	2.18*	− 3.29 ns	− 1.13 ns	12.71 ns	− 1.36 ns	− 0.69 ns	− 1.3**	− 3.24**
CMS-HAP-111 × RHP-53	1.6 ns	1.43 ns	0.91 ns	1.98*	− 11.69 ns	− 2.78 ns	− 3.53*	− 0.61 ns	3.6**
CMS-HAP-111 × RHP-71	1.43 ns	2.43*	0.28 ns	− 0.04 ns	5.29 ns	1.91 ns	− 0.03 ns	− 0.87 ns	− 1.9 ns
CMS-HAP-111 × RHP-69	2.35**	2.26*	− 0.81 ns	1.63 ns	− 6.44 ns	− 4.42 ns	2.25 ns	− 0.42 ns	1.51 ns
CMS-HAP-112 × RHP-68	− 2.07*	− 3.24**	8.99 ns	− 1.01 ns	− 5.9 ns	− 2.51 ns	− 1.39 ns	− 0.07 ns	− 1.65 ns
CMS-HAP-112 × RHP-41	− 2.9**	− 3.15**	− 17 ns	0.13 ns	− 3.4 ns	1.37 ns	− 1.5 ns	− 0.45 ns	2.43*
CMS-HAP-112 × RHP-38	− 2.07*	− 1.82 ns	− 13.28 ns	− 1.47 ns	− 6.65 ns	6.53 ns	− 0.28 ns	0.44 ns	− 1.82 ns
CMS-HAP-112 × RHP-53	− 1.15 ns	− 1.24 ns	1.36 ns	− 0.96 ns	1.64 ns	− 1.29 ns	0.78 ns	− 0.31 ns	2.93**
CMS-HAP-112 × RHP-71	− 0.82 ns	0.1 ns	− 14.81 ns	0.68 ns	15.6*	− 1.03 ns	− 0.25 ns	0.27 ns	− 2.49*
CMS-HAP-112 × RHP-69	− 2.07*	− 1.07 ns	− 6.26 ns	− 0.49 ns	− 1.11 ns	− 1.72 ns	0.86 ns	− 0.44 ns	2.6*
CMS-HAP-12 × RHP-68	3.18**	3.6**	− 7.42 ns	2.08*	11.66 ns	2.5 ns	− 1 ns	0.3 ns	− 2.74**
CMS-HAP-12 × RHP-41	2.6**	1.93 ns	− 1.7 ns	0.61 ns	− 5.24 ns	0.18 ns	− 1.61 ns	0.16 ns	0.26 ns
CMS-HAP-12 × RHP-38	3.01**	2.26*	7.82 ns	2.46*	5.85 ns	− 3.91 ns	0.78 ns	0.64 ns	0.1 ns
CMS-HAP-12 × RHP-53	2.6**	2.93**	− 0.35 ns	0.26 ns	0.95 ns	2.61 ns	2.22 ns	− 0.11 ns	2.26*
CMS-HAP-12 × RHP-71	0.18 ns	− 1.07 ns	− 4.31 ns	− 0.46 ns	− 15.87*	− 5.5 ns	− 0.72 ns	− 0.1 ns	− 0.9 ns
CMS-HAP-12 × RHP-69	0.35 ns	0.26 ns	7.07 ns	1.11 ns	6.42 ns	− 0.31 ns	− 1.17 ns	0.27 ns	− 0.9 ns
CMS-HAP-54 × RHP-68	0.51 ns	1.35 ns	10.85 ns	− 1.02 ns	− 15.4 ns	− 11.96*	− 2.17 ns	− 0.03 ns	1.26 ns
CMS-HAP-54 × RHP-41	0.01 ns	− 0.24 ns	20.26 ns	− 0.11 ns	2.44 ns	− 1.19 ns	− 2.56 ns	− 0.11 ns	2.18*
CMS-HAP-54 × RHP-38	− 1.32 ns	− 2.49*	20.87 ns	− 0.14 ns	− 10.3 ns	1.13 ns	1.28 ns	0.61 ns	− 1.24 ns
CMS-HAP-54 × RHP-53	− 1.32 ns	− 1.9 ns	3.45 ns	− 0.28 ns	− 4.18 ns	− 8.52 ns	− 1.67 ns	0.24 ns	1.93 ns
CMS-HAP-54 × RHP-71	− 0.24 ns	− 0.82 ns	3.09 ns	− 0.5 ns	3.43 ns	5.7 ns	− 0.69 ns	− 0.43 ns	− 0.82 ns
CMS-HAP-54 × RHP-69	− 0.9 ns	− 0.9 ns	1.84 ns	− 0.48 ns	0.42 ns	− 0.66 ns	− 0.36 ns	0.43 ns	1.51 ns
CMS-HAP-99 × RHP-68	− 0.07 ns	− 0.9 ns	− 11.62 ns	0.14 ns	8.38 ns	10.84 ns	− 0.36 ns	− 0.38 ns	− 0.9 ns
CMS-HAP-99 × RHP-41	0.51 ns	2.6*	18.22 ns	0.75 ns	6.97 ns	− 1.16 ns	3.47*	− 0.02 ns	2.26*
CMS-HAP-99 × RHP-38	0.43 ns	1.51 ns	0.18 ns	1.26 ns	− 3.95 ns	− 12.45*	0.58 ns	− 0.21 ns	− 1.07 ns
CMS-HAP-99 × RHP-53	0.68 ns	1.51 ns	7.2 ns	− 0.47 ns	7.62 ns	9.84 ns	3.11 ns	0.12 ns	− 1.07 ns
CMS-HAP-99 × RHP-71	− 1.32 ns	− 1.9 ns	5.91 ns	0.41 ns	− 11.21 ns	1.59 ns	0.17 ns	0.7 ns	1.35 ns
CMS-HAP-99 × RHP-69	1.35 ns	0.6 ns	10.11 ns	− 1.17 ns	− 1.19 ns	8.14 ns	− 2.06 ns	0.03 ns	0.6 ns

*DFI* days to flower initiation, *DFC* days to flower completion, *PH* pant height, *LA* leaf area, *HD* head diameter, *S.C* stem curvatures, *LP* leaves per plant, *HSW* 100 seed weight, *SYP* seed yield per plant.

## Discussion

Moder day agriculture more concerned with enhanced production capacity of crops in combination with efficient utilization of renewable and non-renewable resources^21^. Information and extent of genetic diversity available in a crop is the basic and utmost requirement for developing and designing a hybrid or cultivar improvement program of any crop including sunflower. In the present study, a novel approach of identification of diversity, then a methodology of utilization of the diversity for sunflower hybrid development has been proposed. Clustering is a type of unsupervised machine learning approach that tends to group data points having commonalities in a particular group, while data point in different groups have less similarities^22^. There are various types of clustering algorithms, among them Hierarchical clustering algorithm (HCA) is very common. This clustering technique tends to build a hierarchy of clusters one after the other^23,24^. A hierarchical clustering approach is frequently used in plant sciences for classification and diversity analysis. Use of this machine learning model has been successfully applied for identification of Cysteine-rich Receptor-like Kinase (CRK) genes in *Arabidopsis thaliana*^25^. Likewise, diversity paneling of wheat genotypes has been successfully carried out using HCA^26^. In current study, HCA applied on the sunflower data set, which is a combination of morphological, biochemical, and molecular attributes, to find the optimum number of clusters and most suitable genotype, which would represent the whole cluster in the crossing scheme.

In the case of current study, 2 major clusters are identified by applying the HCA, which could be divided into 6 smaller cluster each (Fig. 1). It was noted that in one major cluster, there were only restorer lines, while the other major cluster contains A-lines, B-lines and SFP line combined. This trend of clear separation of restorer lines from A, B and SFP lines had previously been monitored in sunflower^12,14,27^. Efficiency of HCA has been well documented in diversity paneling. Clustering of barley genotypes using HCA approach was found to be quite successful in delineating genetic diversity analysis^28^. In current study, HCA approach was the most successful in not only separation of R-lines from the rest of genetic materials but also dividing the genotypes into six smaller groups each of major cluster, comprising 12 overall heterotic groups in sunflower genotypes. These high-resolution heterotic groups were in-fact the product of combining different levels of diversity organization in sunflower plants, from molecular to proteins and then to organ and individual level.

K-means clustering (KMC) is another type of clustering/classification approach applied in machine learning, wherein a dataset is classified into a certain k-number of clusters, where k is an integer^29^. Use of KMC is well documented in datasets where the sole objective is to classify a dataset into different groups. The number of k-clusters was identified through hit and trial method. In the present study, the optimum number of clusters was identified at k = 2 at which genotypes can be grouped into two major clusters as observed through, HMC approach. In KMC, restorer lines were grouped separately from the rest of sunflower genotypes under study, however, using KMC approach it is almost impossible to further classify the sunflower lines in smaller clusters for making more accurate identification of potential parents for sunflower hybridization program.

Previously, KMC has been applied to compare gene expression patterns in plants under normal and stressful conditions^30^. Likewise, application of KMC based machine learning approach has been found very informative in functional association of biotic and abiotic genes^31,32^. Use of KMC in agro-morphological dataset of mung bean, revealed that genotypes grouped into seven different clusters irrespective of their geographical origin^33^. Iranian *Rhabdosciadium aucheri*, specie gene-pool were successfully characterized and differentiated into three populations after application of KMC. Hence, usage of KMC based approach is an effective technique for population identification/grouping, however, accurate identification of heterotic grouping and superior potential parents for breeding programs is not possible through KMC based clustering.

In unsupervised machine learning, both hierarchical and K-means clustering approaches utilization are well documented in analyzing unstructured datasets. However, both have their own advantages and disadvantages as well. Hierarchical clustering algorithm cannot represent distinct clusters with similar expression patterns. Moreover, as the size of cluster increases, the actual expression patterns become less relevant. Whereas K-means required a specific k-clusters (k is any integer) in advance to classify dataset into groups, also this algorithm is very sensitive to outliers as well^34^. In contrast to hybrid algorithms combine the strengths of other algorithms and tend to produce much more refined results. Using a hybrid algorithm of k-means and hierarchical clusters, produces better results than the standard average for Euclidean distance for hierarchical clustering. Similarly, much refined results of microarray datasets were obtained using hierarchical and k-means hybrid clusters^35^.

In the present study, a hybrid approach of bagging both hierarchical and k-means clustering was obtained on the combined dataset of morphological, biochemical, and molecular characterization of sunflower. No previous usage of this strategy in sunflower was obtained, making it a unique methodology to study characterization of un-structured dataset through multivariate and unsupervised machine learning techniques. Use of hybrid clusters making k-clusters to 12, as obtained in hierarchical produced 12 clusters of 109 sunflower datasets (Fig. 3). However, using hybrid approach was not as successful as hierarchical clusters alone, as in some cases, A-lines also has been classified with Restorer lines, which was not observed in hierarchical or k-means clustering approach. Therefore, it was deduced that more work on hybrid algorithms by applying other bagging and optimization techniques or use of more than two clusters in hybrid would have been practiced to obtained much better resolution of genotypes in heterotic groups.

Heterosis is defined as deviation observed in means of the progeny as compared to their parents. To exploit heterosis successfully in crop plants, presence of genetic variability among the participating parents is a pre-requisite. Many of the times, positive heterosis or hybrid vigor of F_1_ over their parents or better parent is required as in case of seed yield, 100 seed weight etc. However, in a few cases negative heterosis is also required for some important traits i.e., flowering time, time taken to maturity and plant height in sunflower^36^. In this study, mid and better parent heterosis estimates of 36 sunflower hybrids developed from 12 selected parental lines (each line representing a specific heterotic group) showed that both negative and positive heterotic values were obtained for different nine highly important plant characteristics.

Out of 36 F_1_ crosses tested for heterosis and heterobeltiosis showed that majority of F_1_ hybrids have shown heterosis in the desirable direction for all the traits under consideration. For days to flower initiation, days to complete flowering and plant height, most of the hybrids expressed a negative heterosis and heterobeltiosis effects as compared to their parents. Likewise, for leaf area and head diameter both positive and negative heterotic effects were observed, while for stem curvature, number of leaves per plant, 100 seed weight and seed yield per plant majority of the F_1_ hybrids under examination expressed a positive heterotic value against mid-parent and better parent means.

Desirability of heterotic direction depends upon the overall contribution of the component traits towards seed yield or oil yield in sunflower. As negative heterosis is desired in sunflower for flowering traits because the plants that are early in starting their flowering stage will have more time left to remain in the field for grain filling stages, thus a negative heterosis for flowering traits will ultimately lead to high seed yield in sunflower^37,38^. Likewise, leaf area corresponds to the availability of photosynthetic surfaces, therefore, heterosis in positive direction is required^37^. Similarly, head diameter is directly proportional to the surface available for seed filling, hence increase in head diameter over parental lines is desirable in sunflower breeding program^39,40^.

Since leaf area is the target of photosynthesis, previous results indicate that positive heterotic values are required for sunflower leaf area. Negative and positive heterosis, and heterobertiosis values are found regarding the leaf area in the present experimentation and these findings are also supported by Khan et al.^35^. Furthermore, Habib et al.^41^ and Khan^42^ confirmed that a higher positive vigor per 100 seeds is required as it is directly linked with economical yield of sunflower crop. Regarding our findings, 34 out of 36 hybrids showed a positive heterosis effect, likewise 23 hybrids were also positive in case of heterobeltiotic effect depicting that these hybrids had higher test weight as compared to both parents. Previous studies on sunflower heterosis estimation also confirmed that 100 seed weight increases as the distantly related genotypes crossed to produced F_1_ hybrids in sunflower^37,39,40,43^. Many researchers like Kaur^40^, Radhika et al.^39^, Phad et al.^43^, Alone et al.^44^, Manivannan et al.^45^, Sawant et al.^46^ and Channamma^47^ reported significant heterotic effect of seed yield in hybrids developed experimentally using diverse male and female lines.

It is generally believed that parents having high combining ability are not able to transmit their high yield potential to their progeny, hence estimation of combining ability is a pre-requisite for developing a high yielding and sustainable hybrids or cultivars. To increase the yield in sunflower vertically, development of hybrids with better yield potential and stability is required. Parents with diverse genetic makeup would generally produce superior transgressive hybrids^48^. To make a breeding program fruitful, the first step is to select the parental lines to be used for hybridization. In crop plants including sunflower, genetic variability, type of gene actions and combining ability analysis are the most important parameters^49^.

The occurrence of both significant GCA and SCA effects in the present study indicates the presence of both additive and non-additive gene effects in the expression of plant measured traits. GCA effects generally lead to the selection of suitable parents for population improvement or development of synthetics or composite cultivars, as these may be the preference of some growers because their seed can be used for more than one year^48^. In present study, high and significant GCA effects for both among CMS and restore lines in desirable directions i.e., negative for flowering, maturity, and plant height traits and positive for the rest of traits measured was observed. Short duration varieties are the preference of sunflower growers as these can reduce the risk of exposure to adverse climatic and biotic factors like diseases and insect attack^48^. Development of high yield hybrid/cultivar along with shorter growing period is among the prime objectives of sunflower breeder(s)^49^.

In present study, higher magnitude of SCA effect than that of GCA was observed for days to flower initiation, days taken to flower completion, head diameter, plant height, leaf area and 100 seed weight suggesting a pre-dominance of SCA/non-additive factors in controlling these flowering and yield affecting traits in sunflower. Control of these sunflower plant traits through a dominant or epistatic type of gene actions has been previously reported in many studies^40,50,51^. While a higher magnitude of GCA effects was recorded for stem curvature, number of leaves per plant and seed yield per plant. These higher values of GCA than SCA showed that genes controlling these traits are having an additive type of genetic inheritance and therefore the parental lines to be used for crossing programs must be improved first or should have high potential for these traits before using them in sunflower hybrid breeding program.

## Conclusion

Application of machine learning in plant improvement programs would become a vital tool for breeders as it can speed up the steps involved in the release of final cultivar for general cultivation. However, more efforts for optimization and accurate application of machine learning algorithms in plant breeding is needed. In this study, two unsupervised machine learning clustering algorithms, i.e., hierarchical and k-means were applied on a combined morphological, bio-chemical, and molecular dataset of sunflower genotypes. In addition, a hybrid cluster algorithm of hierarchical + k-means was also designed and implanted on the same dataset for heterotic grouping identification. Results showed that hierarchical clustering approach is more suitable in given circumstances. Hence, 12 heterotic groups were identified (6 for CMS lines and 6 for restore lines), and one genotype from each group was selected as a representative of whole identified group. Selected 12 lines (one each from each heterotic group) were crossed in a L × T design and resulting F1 were evaluated in open field conditions for combining ability and heterosis studied. Results showed that most of the hybrid developed exhibited a significant amount of heterosis for all the studied traits and more importantly in the desirable directions. However, three hybrids (1) RHP-41 × CMS-HAP-56, (2) RHP-71 × CMS-HAP-111 and (3) RHP-71 × CMS-HAP-12 are more suitable for further evaluation and release of new sunflower hybrid cultivar.

### Supplementary Information

Supplementary Information 1.

Supplementary Information 2.

## Data Availability

The data that support the outcomes of the current experimentation are available from the corresponding author upon reasonable request.
