# Supplementary material for: Application of machine learning for identification of heterotic groups in sunflower through combined approach of phenotyping, genotyping and protein profiling
Source: Sci Rep. 2024 Mar 27;14:7333. doi: 10.1038/s41598-024-58049-z (PMC10973396; doi:10.1038/s41598-024-58049-z)
Supplement: Supplementary file 2 — Supplementary Information 2. [file 41598_2024_58049_MOESM2_ESM.docx]

**Table S2.** List of SSR markers used to study the genotypic diversity in 109 sunflower genotypes

| Primer Name | Linkage Group | Forward Sequence | Reverse Sequence |
| --- | --- | --- | --- |
| ORS-605 | 1 | CGCGTGATGTGACGATTATT | ACGGAGCAAAGTTTCGAGGT |
| ORS-543 | 1 | CCAAGTTTCAGTTACAATCCATGA | GGTCATTAGGAGTTTGGGATCA |
| ORS-371 | 1 | CACACCACCAAACATCAACC | GGTGCCTTCTCTTCCTTGTG |
| ORS-453 | 2 | CCTGTGAGCTACAATACTCCCACA | GATTCTGATTAGGCGGTGGT |
| ORS-1053 | 2 | TTTCATCACATTAGACCATAGACCA | GGCTTTCCTTCGTGGTTTGTAT |
| ORS-752 | 3 | CACTGATGAACAAGTGCGAGA | ATGATTCCCATACCCACCAA |
| ORS-924 | 3 | TAAATCGCCATACCACTCCATC | TATCAGCAGGAAGAACGCCTAAT |
| ORS-366 | 4 | AACCAACTGAGCATTCTTGTGA | GCGCTAGGTTAAAGAGGACAAA |
| ORS-1068 | 4 | AATTTGTCGACGGTGACGATAG | TTTTGTCATTTCATTACCCAAGG |
| ORS-337 | 4 | TTGGTTCATTCATCCTTGGTC | GGGTTGGTGGTTAATTCGTC |
| ORS-1024 | 5 | GGGAAGTGGGCTTGTCTATGTAT | AACACACCGAAATCACCTATGAA |
| ORS-533 | 5 | TGGTGGAGGTCACTATTGGA | AGGAAAGAAGGAAGCCGAGA |
| ORS-608 | 6 | CATGGAAAGCCGAGTTCTCT | CGTGCGTGATTAACATACCC |
| ORS-1256 | 6 | GATGTTGATGTTGGTGAAGTTGC | CTCCGTCACCTTAAGCACTTGTA |
| ORS-400 | 7 | CGAACCCGTCTGTACCGTTT | ACTTCGTTCACAAGGCACAA |
| ORS-700 | 7 | GTACCCACCACGCTTAACCA | AGTCTTCCACAGCAACGTCA |
| ORS-830 | 8 | CAAGTGCATTAGGTGGTTCTAACA | GCCCTCTGACTGTTGTATGACTG |
| ORS-599 | 8 | TTCCCTATCACACGCCTCTC | GAAAGGAAGTAGCGGTGGTG |
| ORS-882 | 9 | AAACCGGCATGTAAGATATTCG | ATCGGGAGCAGAAGAAGAGTATG |
| ORS-617 | 9 | GGTACTTGGTATTCATGGGTCAT | GACACCGCCAACTTAACACTT |
| ORS-795 | 9 | CGCTAGTTACACCGCAGATG | TGTCCACAGGTTGAAGATCG |
| ORS-613 | 10 | GTAAACCCTAGGTCAATTTGCAG | ATCTCCGGAAAACATTCTCG |
| ORS-1088 | 10 | ACTATCGAACCTCCCTCCAAAC | GGATTTCTTTCATCTTTGTGGTG |
| ORS-433 | 10 | CCGAGGTTTGATCGCTATTT | AGCGTTTGTGATTTGATTACGA |
| ORS-769 | 11 | GTTTATTTATGTAGAAATGTTCTGGAA | ATGTGGTGGTAAGGGTTGTTG |
| ORS-697 | 11 | TTGGGCTGTGGTTCCTTAAC | AAGAGATGGGAGTGTTGATGC |
| ORS-1085 | 12 | GACCTCAAGGCATGCTAACACTC | ACTAAGTGTGTGGACGGGGAAA |
| ORS-1040 | 12 | CTGCTGATCGTTTCTTGGATAGA | TGCTAATCCTTCTAATCAACTTCCAC |
| ORS-879 | 13 | GAACCTCCCTTTGTCTGCATATC | CTCCGGTTGCTGTTGATGTCT |
| ORS-781 | 13 | GTCAACCCATGACCCAAACC | GATGTGGAGGAGAGAGGGTGT |
| ORS-511 | 13 | TGGCTCAGATTAAGTTCACACAG | CGGGTTGCGAGTAACAGGTA |
| ORS-307 | 14 | CAGTTCCCTGAAACCAATTCA | GCAGTAGAAGATGACGGGATG |
| ORS-1086 | 14 | TTGTTTGTCGCACACTCAAGATT | ATTATCGGCACATCTTTGGATTT |
| ORS-857 | 15 | ACATCCGAACGAAGGACAATC | CAAGAAAGTATGTCACCCAATAGCA |
| ORS-562 | 15 | CACACACACAAACCCTAGCTCT | CAATCATATCGAGCACACATCA |
| ORS-768 | 16 | CCACTCATCATCAAGCCTAACA | AGGTGGTGCTGGTTGTAGGT |
| ORS-1064 | 16 | TGAATGATCTATGAGTGGTGATGG | ACTCGCAGTGGTAAGTCGTTAGG |
| ORS-495 | 16 | CCAGGATTAGGTAGCTTAGTTCG | GCGATCTGAGGTTGACTCGT |
| ORS-811 | 17 | CCTTCTCCTCAATCTTTGGCTA | AGGAATGAAATGGGTGTGTGT |
| ORS-845 | 17 | GGTGCCCTATCTTCATTCTCTG | CTAAAGGGTATCACACATTTGACATT |
